# Neutralization-Enhancing RF Antibodies for HIV Vaccines

**DOI:** 10.3389/fimmu.2014.00634

**Published:** 2014-12-16

**Authors:** Konstantin V. Suslov

**Affiliations:** ^1^Institute of Immunology, Moscow, Russia

**Keywords:** HIV, gp120 glycoprotein, neutralizing antibodies, rheumatoid factor, neutralization-enhancing RF antibodies, repeated immunization, microspheres, HIV vaccine

Efficient neutralization of HIV is a primary goal both for therapeutic and prophylactic HIV vaccines based on induction of neutralizing antibodies (NAbs) (1, 2). Neutralization capacity of NAbs correlates with their affinity to HIV-1 gp120 envelope glycoprotein (3). In HIV-infected individuals, early IgM antibodies have low affinity for gp120 glycoprotein (3), which is slightly compensated by their pentameric structure and avidity to multivalent structures of the virus. Corresponding IgG1 antibodies (3, 4), which appear later from IgM through the CSR and SHM processes (3, 5), have high affinity and specificity to gp120, but low avidity due to monomeric structure of IgG. The question is how to induce HIV-specific NAbs with both high affinity and avidity to gp120?

Anti-HIV IgG antibodies 2F5 and 2G12, switched back to IgM isotype, showed increased avidity and neutralization efficacy (2). Dendritic antibody supramolecules (DAS) in one molecule combine high specificity of IgG with high avidity of IgM (6). It would be of interest to construct such a DAS with IgM as a core, carrying 10 anti-gp120 IgG monomers. Natural monomeric anti-gp120 IgG1 antibodies (3, 4) not only have low avidity insufficient for effective neutralization of HIV but may also contribute to FcR-mediated infection enhancement (7, 8). Does it mean that HIV may use high-affinity IgG antibodies for its own purposes? Fortunately, in nature there exist some helpful network-like regulatory mechanisms with a key role of RF antibodies (9).

Rheumatoid factor (RF) is an autoantibody which specifically binds Fc region of IgG (10, 11). Apart from pathologic RF in rheumatoid arthritis (12), natural RF appears in many non-rheumatoid states (13). Possible beneficial physiological roles of RF include enhanced clearance of immune complexes (ICs) (14), amplification of IgG response to pathogens (15), and enhancement of virus neutralization (16, 17). RF can be induced *in vivo* in a highly specific way either by secondary immunization with protein antigens or in response to immunization with the newly formed ICs (10, 11). New antigenic determinants, which appear in the Fc region of IgG antibody upon antibody-antigen complex formation, may strengthen the specificity of RF (9).

The level of RF was significantly higher in some of HIV-infected individuals compared to control groups (18–22). RFs were mainly IgA, IgM immunoglobulins with specificity against anti-HIV IgG (20). RF-mediated enhancement of anti-HIV IgG neutralization activity was found in the sera from MCTD patients (17). Authors suggested that RF is promising for passive immunotherapy based on NAbs (17).

Can RF play a key role in specific enhancement of IgG-mediated neutralization of HIV *in vivo*? Measurements of RF level both in long-term non-progressors (23) and dual-infected individuals (24) might give some clues. Repeated immunization of uninfected macaques with HIV-1 gp120 glycoprotein may allow researchers not only to track the kinetics of RF induction but also to elucidate whether neutralization-enhancing RF antibodies can protect macaques against subsequent challenge with SHIV. Repeated immunization of SHIV-infected macaques with HIV-1 gp120 glycoprotein might show whether neutralization-enhancing RF antibodies can prolong the asymptomatic period and delay the onset of AIDS.

The level of RF is not stable and has a tendency to decline during the acute phase of HIV infection (22). Prolonged repeated immunizations with gp120 (e.g., immunizations every 3 weeks; the actual time between immunizations will be adjusted according to measurements of RF level in patients) would be the solution to keep the level of neutralization-enhancing RF antibodies constantly high. Single-administration vaccine (SAV) technology, which is based on pulsatile release of gp120 from biodegradable polymeric microspheres, mimics repeated immunization scheme, and allows vaccination to be done in one shot (25, 26).

Patient-specific therapeutic HIV vaccines (1) even in simplified version, using the only one gp120 variant formed after completion of HIV population homogenization process (27), can be performed via repeated immunizations from biodegradable polymeric microspheres under SAV (25, 26) technology platform. Prophylactic HIV vaccines can be based on the variations of sequential scheme (28) for prolonged pulsatile release of gp120 glycoproteins from biodegradable polymeric microspheres in single-shot (25, 26) way convenient for both patients and doctors.

Studies (15, 17) have shown the high potential of neutralization-enhancing RF antibodies, but several principal questions arise:

(i) Can neutralization-enhancing RF antibodies (NeRFa) be induced after repeated immunization of humans with recombinant gp120 glycoprotein?
(ii) Will the power of gp120 immunogen design (29–31) combined with an optimal vaccination regimen help the induction of NeRFa?
(iii) Could NeRFa help to improve the efficacy of previous (32) and future HIV vaccines based on induction of NAbs?
(iv) Might induction of NeRFa be a future promising method not only against malaria as suggested in Ref. (33), but also against life-threatening viruses like Ebola (34)?

Repeated immunization with gp120 glycoprotein might lead to prolonged induction of neutralization-enhancing RF antibodies with a potential to be explored for finding the ways to extend lives of HIV-infected individuals and to stop current HIV pandemic.

## Conflict of Interest Statement

The author declares that the research was conducted in the absence of any commercial or financial relationships that could be construed as a potential conflict of interest.
